# Major Adverse Cardiovascular Events in Patients with Acute Myocardial Infarction and Angiographic Evidence of Coronary Artery Ectasia: A Systematic Review and Meta-Analysis

**DOI:** 10.3390/jcm15093482

**Published:** 2026-05-01

**Authors:** Nikolaos Otountzidis, Nikolaos Stalikas, Amalia Baroutidou, Efstratios Karagiannidis, Matthaios Didagelos, Barbara Fyntanidou, Antonios Ziakas, George Giannakoulas

**Affiliations:** 1 First Department of Cardiology, AHEPA University Hospital, Aristotle University of Thessaloniki, 54636 Thessaloniki, Greece; nickotountzidis@gmail.com (N.O.); bamalia27@gmail.com (A.B.); manthosdid@yahoo.gr (M.D.); tonyziakas@hotmail.com (A.Z.); 2 Department of Cardiology, Cardiovascular Center OLV Hospital, Moorsellbaan 1654, B-9300 Aalst, Belgium; nstalik@gmail.com; 3 Department of Emergency Medicine, Aristotle University of Thessaloniki, 54636 Thessaloniki, Greece; stratoskarag@gmail.com (E.K.); bfyntan@yahoo.com (B.F.)

**Keywords:** coronary artery ectasia, percutaneous coronary intervention, myocardial infarction

## Abstract

**Background/Objectives:** Coronary artery ectasia (CAE) presents challenges, specifically in the context of percutaneous coronary intervention (PCI), and has been associated with adverse events, particularly in the setting of acute myocardial infarction (AMI). The objective of the present study was to assess whether CAE is associated with increased occurrence of major adverse cardiovascular events (MACEs) in patients with AMI. **Methods:** A systematic review and meta-analysis of observational studies were conducted. We systematically searched MEDLINE via PubMed, Scopus, the Cochrane Library (CENTRAL), ClinicalTrials.gov, and reference lists to identify eligible studies. Baseline characteristics, comorbidities, angiographic data, and rates of MACEs and their individual components (all-cause or cardiovascular mortality, repeat AMI, repeat revascularization, stroke, and heart failure) have been extracted. The results were synthesized as odds ratios (ORs) using random-effects models. **Results:** Ten studies and 13,908 patients were included. CAE was found to be predictive of higher odds of MACEs [OR: 2.12, 95% CI: 1.34 to 3.36]. No difference was found regarding the odds of all-cause and cardiac death. The presence of ectasia was associated with higher odds of recurrent AMI, compared with controls [OR: 2.76, 95% CI:1.62 to 4.71]. The groups were comparable regarding the need for repeat revascularization, while the reports on stroke and heart failure were scarce. **Conclusions:** The results highlight the compounding effect of CAE on future MACE events in patients with AMI. Patients with AMI and CAE have higher odds of repeat AMI compared to patients without CAE, while mortality and repeat revascularization rates are similar. This might indicate the need for more aggressive treatment strategies in these patients.

## 1. Introduction

Coronary artery ectasia (CAE) is a rare anatomical abnormality of the coronary arteries, with an incidence ranging between 0.4% and 4.9%, and is more commonly observed in males [1,2]. It represents an abnormal focal or diffuse dilation of any coronary artery segment of at least 1.5 times the adjacent normal vessel [1].

Genetic factors, progressive atherosclerosis, and vascular inflammatory disease have all been associated with CAE, though its exact pathogenesis remains elusive. Most patients with CAE are asymptomatic, and thus the diagnosis is often incidental during coronary angiography or coronary computed tomography angiography (CCTA) performed for various reasons [3]. However, CAE may be an angiographic finding in patients presenting with acute myocardial infarction (AMI) and can pose various treatment challenges and periprocedural complications [4]. The aneurysmal nature of the culprit artery predisposes the formation of an increased thrombus burden. The restoration of thrombolysis in myocardial infarction (TIMI) III flow may necessitate thrombus aspiration techniques and/or the use of glycoprotein IIb-IIIa antiplatelet treatment, increasing the risk of periprocedural complications [5,6]. Additionally, proximal and distal landing zones are usually unclear, and potential stent undersizing increases the risk of stent thrombosis and migration. Indeed, a higher failure rate of PCI has been reported in patients with CAE compared to patients without CAE [7]. However, due to contradictory results regarding major adverse cardiovascular events (MACEs) in patients with CAE presenting with AMI [7,8,9,10,11,12], clinical guidelines on the management of ectatic vessels, in the setting of AMI, remain scarce [13,14]. Moreover, previous meta-analyses, limited by a smaller sample size, failed to demonstrate a significant association between CAE and MACEs [15].

The objective of this study was to systematically review the existing literature and investigate whether patients with AMI and documented CAE have an increased risk of MACEs compared to patients without evidence of coronary ectasia.

## 2. Materials and Methods

The present systematic review was conducted in accordance with the Preferred Reporting Items for Systematic Reviews and Meta-Analysis (PRISMA) guidelines [16]. The protocol describing the methods of analysis and the inclusion criteria for the present systematic review has been registered in advance with the International Prospective Register of Systematic Review (PROSPERO), registration number CRD42022369673, available from: https://www.crd.york.ac.uk/prospero/display_record.php?ID=CRD42022369673 (website last accessed on 15 December 2025).

### 2.1. Eligibility Criteria

In the present systematic review, prospective/retrospective cohort studies and case–control studies were included. Case reports/series and reviews were excluded. No restrictions were imposed regarding the language, the publication date, or the status of the studies. All included patients were over 18 years of age and underwent coronary angiography (CA) after AMI, including ST-elevation myocardial infarction (STEMI) and non-ST-elevation myocardial infarction (NSTEMI) [13,14]. Studies enrolling patients undergoing CCTA or coronary magnetic resonance angiography were excluded. Moreover, studies that included cases in which CA was performed for reasons other than AMI (e.g., stable/unstable angina) were excluded. 

Patients with AMI and evidence of CAE (focal or diffuse dilation of the coronary artery segment of at least 1.5 times the adjacent normal artery) in the CA were included as the exposure group. The outcomes were compared to those of patients with AMI and without angiographic evidence of CAE. Studies on CAE only in the infarct-related artery were included, on the supposition that patients with CAE in other arteries were excluded. Stent-related or other defined iatrogenic coronary aneurysms were excluded.

The primary outcome of the present study was the composite outcome of major adverse cardiovascular events (MACEs), including all-cause mortality or cardiac death, repeat AMI, repeat revascularization, stroke, and hospitalization due to heart failure. Due to the possibly variable definitions of the term MACE, any study reporting MACEs as a composite outcome of at least two of the individual components of the term was considered a MACE reporting study. The individual components of MACEs (all-cause death, cardiac death, repeat AMI, repeat revascularization, stroke, and hospitalization due to heart failure) were studied as secondary outcomes. Focusing on studying the long-term outcomes of patients with CAE after AMI, we chose to include studies with a follow-up period for the reported outcomes of at least 6 months post-AMI.

### 2.2. Search Strategy

Relevant studies were identified via a systematic search of the literature in various electronic databases. In detail, we searched the National Library of Medicine’s MEDLINE database via PubMed, Scopus by Elsevier, and the Cochrane Library, CENTRAL. Moreover, ClinicalTrials.gov was assessed to identify relevant, yet unpublished, ongoing trials. The reference lists of the included studies have been hand-searched for any unidentified papers from the initial search. Finally, the PROSPERO database was searched to identify relevant studies to ensure that there are no other ongoing systematic reviews on the present subject.

Systematic research was performed in each of the above-mentioned databases, using a predefined search strategy with terms regarding the studied population (Supplementary File, Supplementary S1–S4). No language restriction or other search filters were applied in any of the databases. All searches were completed in December 2025.

The quality of the above-mentioned search strategy was evaluated by the researchers, using the Evidence Based Checklist for the Peer Review of Electronic Search Strategies (PRESS EBC) [17].

### 2.3. Selection Process

The search results from the above-mentioned databases were inserted into the automation tool “Deduplicator” in the Systematic Review Accelerator website by Bond University for the removal of duplicate records of the same reports [18]. The titles and abstracts of the deduplicated studies were then screened in a blinded manner by two independent authors (N.O. and N.S.), using the automation tool “Screenatron” in the Systematic Review Accelerator [19]. Any disagreements were solved by a third researcher (A.B.). The full-text articles from the reports eligible by initial screening were sought for retrieval. All full texts were retrieved and assessed for potential inclusion in the study. No multiple reports of the same study were identified after juxtaposing author names, comparisons, sample sizes, and outcomes. The full-text screening was performed by two independent authors (N.O. and N.S.). In this step of screening, the reason for the exclusion of each study was reported.

### 2.4. Data Collection Process

The data extraction process was performed by two authors via predefined forms. The forms were pilot tested in three random reports of the selected studies to ensure all relevant information was included. The whole process was performed using the Covidence™ software (Covidence systematic review software, Veritas Health Innovation, Melbourne, Australia, available at www.covidence.org, last accessed on 16 January 2026).

The extracted data included study identification and characteristics, patients’ characteristics, comorbidities, primary and secondary outcomes.

### 2.5. Study Risk of Bias Assessment

The quality of the included observational cohort studies was assessed using the Newcastle–Ottawa Quality Assessment Scale (NOS) for Observational Studies [19]. Τhe NOS utilizes a star system to evaluate the quality of a study in three domains, including the selection of the study groups, the comparability of these groups, and the ascertainment of the outcome of interest for cohort studies. A study can be given a maximum of one star for items within the selection and outcome categories, while a maximum of two stars can be awarded in the comparability section, resulting in a total maximum of nine stars. Quality assessment was performed separately for each reported outcome by two authors (N.O. and N.S.).

### 2.6. Synthesis Methods

Data on normally distributed continuous variables were reported as the mean ± standard deviation, while non-normally distributed variables were reported as the median with interquartile range. Categorical variables were reported as frequencies and percentages. The odds ratio (OR) was used as the summary measure for the outcomes of interest, calculated by the reported frequencies in each study. Given that the included studies were sufficiently homogeneous regarding their design and their reported outcomes, a meta-analysis was performed. The ORs from the included studies were combined using a Mantel–Haenszel formula, assuming a random-effects model, with the DerSimonian–Laird estimator for between-study variance. The results were presented using forest plots.

- Statistical heterogeneity between the studies was estimated via the Cochran Chi-squared (χ^2^) test and the I^2^ statistic. I^2^ values larger than 75% were thought suggestive of considerable heterogeneity, and in those cases, a meta-analysis was not performed.
- Due to the small number of studies reporting each outcome, a subgroup analysis was not considered appropriate. Moreover, as all the included studies showed a similar level of quality, a sensitivity analysis considering study quality was not performed. However, post hoc sensitivity analyses were conducted to explore heterogeneity between studies.
- All statistical analyses were performed using the RevMan 5.4 statistical software (Review Manager, Version 5.4, The Cochrane Collaboration, 2020). The analyses were performed at a 0.05 significance level.

### 2.7. Publication Bias Assessment

For the assessment of the possible effect of publication bias on the results, we visually evaluated the funnel plot of each outcome for asymmetry (Supplementary Figures S1–S6). The use of formal tests for asymmetry (Egger’s test) was avoided, as all our outcomes included fewer than ten studies.

## 3. Results

### 3.1. Study Selection

**Figure 1:**
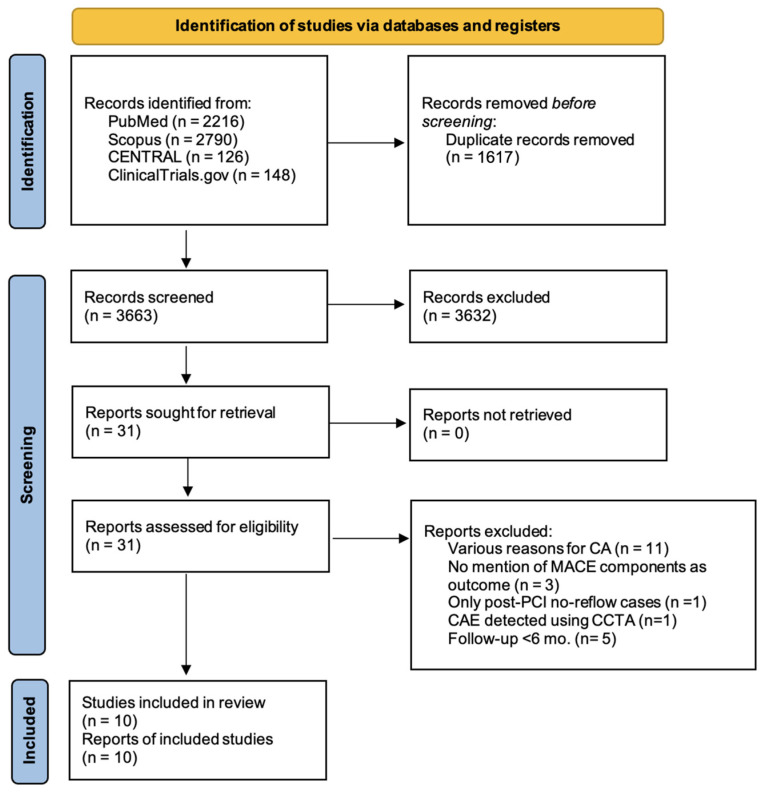
A PRISMA 2020 flow diagram depicting the study selection process. CA: coronary angiography, MACEs: major adverse cardiovascular events, PCI: percutaneous coronary intervention, CAE: coronary artery ectasia, and CCTA: coronary computed tomography angiography. From: [16].

The search strategy resulted in 2216 articles from PubMed, 2790 articles from Scopus, 126 articles from the Cochrane Library, and, finally, 148 articles from ClinicalTrials.gov. No additional studies, unmarked from the initial search, were found during the hand-search of the reference lists of included studies. After removal of duplicates, a total of 3663 studies remained. During the title and abstract screening, 3632 studies were excluded, resulting in 31 papers for the final full-text screening. Ultimately, 10 studies, recruiting a total of 13,908 patients, met the inclusion criteria. The search results are presented in detail in the PRISMA flow diagram (Figure 1). 

### 3.2. Study Characteristics

Nine of the included studies were retrospective cohorts, while only one of the cohorts (Iannopoulo et al.) was prospective [10,11,12,20,21,22,23,24,25,26]. All cohorts were single-centered. CAE in all studies was defined as focal or diffuse dilation of the coronary artery segment of at least 1.5 times the adjacent normal artery. The mean follow-up period ranged between 12 and 60 months.

The studies included a total of 13,908 adult patients, 689 in the CAE group and 13,219 in the no-CAE group. Four of the cohorts included a predefined ratio of patients in the control group (no-CAE) for the analysis, either matched for baseline characteristics or randomly assigned [10,20,22,26]. The rest of the studies enrolled consecutive patients presenting with AMI. Most studies included only STEMI patients, and in only three studies did the population consist of both patients with STEMI and NSTEMI. Four of the cohorts included in the study group included solely patients with infarct-related CAE [11,20,23,25]. In these cases, patients with CAE in other vessels were excluded from the control group. However, two studies did not define the relation of the included CAE cases with the infarct-related artery [10,26]. The characteristics of the included studies are summarized in Table 1.

The patients included in the present systematic review were mostly males, with a frequency ranging from 80.4% to 97.7% in the CAE group and 71.2% to 97.5% in the no-CAE group. Four studies reported a significantly higher proportion of male patients in the CAE group [21,22,24,26]. The mean age of the patients with CAE ranged between 52.8 and 65.9 years, while the age of patients without ectasia was between 56.5 and 68.0 years. Two cohorts reported significant differences in patients’ age between groups, including younger individuals in the study group [21,23].

In all studies except the one by Iannopolo et al., patients with CAE had a lower incidence of diabetes [10,12,20,22]. Hypertension was similar between the groups, except for the study by Ipek et al., which reported significantly lower rates of hypertension in the CAE group. The same pattern applied to patients with dyslipidaemia, with comparability between the groups in all but two studies [20,22]. In most studies (seven studies), patients with CAE had a higher percentage of smokers compared to the control group [10,11,21,22,23,24,26]. All baseline characteristics of the included patients are presented in Table 2.

The right coronary artery (RCA) was the vessel presenting more frequently dilatation, with frequencies ranging from 45.5% to 79.3%. Four studies included patients with evidence of CAE only in the infarct-related artery [11,20,23,25]. In the study by Iannopollo et al., the RCA was more frequently the culprit vessel in cases where ectasia was present, compared to patients without CAE (*p* = 0.035) [25]. Similar angiographic evidence was also present in the study by Fujii et al. In 69.2% of patients with STEMI, the culprit lesion was present in the ectatic RCA, while in the patients without CAE, the RCA was affected in 38.7% of the cases (*p* = 0.0029) [23]. The angiographic characteristics are presented in Table 3.

### 3.3. Risk of Bias in Studies

The risk of bias assessment was performed by utilizing the Newcastle–Ottawa Quality assessment scale for cohort studies (Supplementary Table S1). The assessment was performed in an outcome-specific manner, and the included studies depicted overall a moderate level of methodological quality, with, however, substantial risk of selection bias and residual confounding, given the retrospective and single-center design of most studies. No points were lost from any study in the selection section, reflecting a great representativeness of the cohorts, a secure assessment of the exposure (CAE), and a clear demonstration that the outcome was not present at the beginning of the study. Given that only three studies were matched for baseline characteristics, the rest of the studies failed to achieve a star rating regarding the comparability of the cohorts [10,20,26]. Finally, in the outcome section, all studies were awarded the stars that correspond to the outcome assessment and the duration of the follow-up, as the outcome was confirmed by reference to medical records, and the follow-up of more than 6 months was considered adequate for the studied outcomes. Most of the included cohorts (seven out of 10) succeeded in completing the follow-up period for all the subjects. In general, there were no variations between the reporting quality of different outcomes in any study.

### 3.4. Major Adverse Cardiovascular Events

MACEs were reported as a composite primary or secondary outcome in seven of the included studies [10,12,20,21,24,25,26]. Only the study by Djohan et al. included all the individual outcomes defined in the present review, namely, all-cause mortality, repeat AMI, repeat revascularization, stroke, and heart failure [21]. All-cause mortality [11,12,20,22,23,25], cardiac death [10,21,23,24,25,26], and repeat AMI [12,21,22,24,25,26] were reported as separate outcomes in six studies. Although AMI was part of the MACE components, Boles et al. and Bogana Shanmugam et al. did not report the individual rates of repeat myocardial infarction [10,20]. The need for repeat coronary revascularization was stated in four studies [11,12,24,26], while only three studies reported the rate of stroke occurrence between the two groups [12,24,26]. Lastly, only the study by Djohan et al. examined if CAE was associated with a higher frequency of new heart failure presentation [12]. The primary and secondary outcomes of the included studies are summarized in Supplementary Table S2.

**Figure 2:**
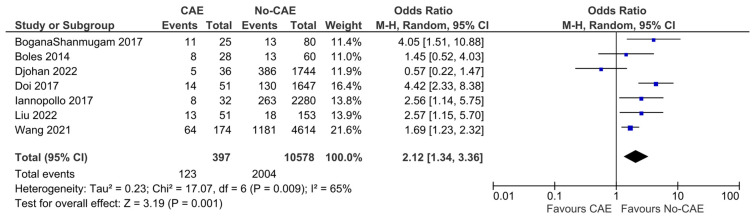
Forest plot of odds ratios (ORs) for additional MACEs in patients with AMI and evidence of CAE versus No-CAE. Results from Mantel–Haenszel (M-H) random-effects meta-analysis, comparing patients with CAE versus patients without CAE. CIs = Confidence intervals [10,12,20,21,24,25,26].

**Figure 3:**
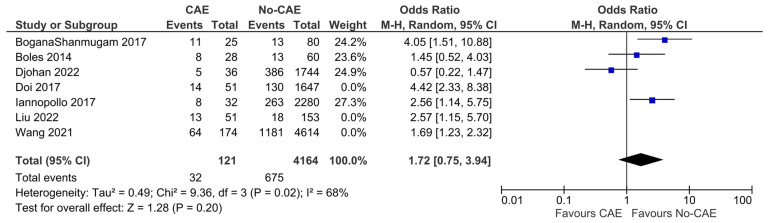
Post hoc sensitivity analysis on additional MACE odds, including studies that enrolled only patients with STEMI. Results from Mantel–Haenszel (M-H) random-effects meta-analysis, comparing patients with CAE versus patients without CAE. CIs = Confidence intervals [10,12,20,21,24,25,26].

The definition of MACEs as composite adverse events varied considerably across these studies. However, the pooled analysis showed significantly higher odds of MACEs in patients with CAE compared to patients without evidence of CAE (OR: 2.12, 95% CI: 1.34 to 3.36, *p* = 0.001). The presence of CAE was associated with an absolute risk increase of 14.2% (95% CI: 4.9% to 25.0%), corresponding to a number needed to harm (NNH) of 7 (95% CI: 4 to 20). These results present significant evidence of heterogeneity (χ^2^ = 17.07, I^2^= 65%) (Figure 2). In the post hoc sensitivity analysis restricted to studies enrolling only STEMI patients, the pooled outcomes differed from the primary analysis, without improvement in heterogeneity (OR: 1.72, 95% CI: 0.75 to 3.94, *p* = 0.2, χ^2^ =9.36, I^2^= 68%) (Figure 3).

In the study by Boles et al., patients with CAE did not show an increased rate of MACEs (ACS, AMI, or cardiac death) compared to patients without CAE (*p* = 0.08) during a follow-up period of two years [10]. In a case–control study of patients with STEMI, MACE occurrence (all-cause mortality, repeat AMI, unstable angina, and need for CABG) was significantly more common in patients with CAE compared to patients without CAE (44% vs. 16.3%, *p* = 0.01) [20].

In the study by Doi et al., CAE was found to be an independent predictor of MACEs (cardiac death or MI) compared to patients without CAE (aHR: 4.94, 95% CI, 2.3 to 10.4; *p* < 0.001) [21].

In the study by Iannopollo et al., CAE in the infract-related artery was independently associated with an increased occurrence of MACEs (all-cause death and AMI) compared to patients without CAE (aHR: 2.24, 95% CI: 1.0 to 5.4; *p* = 0.04) [25]. Comparable results were found in a similar study by Wang et al. where CAE was independently associated with heightened occurrence of MACEs (cardiac death, AMI, repeat revascularization and stroke) compared to no-CAE in both univariate (HR: 1.55, 95% CI: 1.2 to 2.0, *p* = 0.001) and multivariate analysis (aHR: 1.597, 95% CI: 1.2 to 2.1, *p* < 0.001) [24].

Aligned with previous studies, Liu et al. reported that MACEs (stroke, cardiac death, major hemorrhagic events, malignant arrhythmias, cardiogenic shock, readmission, revascularization therapy, and AMI) were significantly increased in patients with CAE presenting with AMI compared to patients without CAE (37.3% vs. 20.3%, *p* = 0.015) [26].

Finally, contrary to other studies, Djohan et al. showed that MACE occurrence (all-cause mortality, repeat AMI or revascularization, stroke, and heart failure) did not differ among patients with CAE and without CAE presenting with STEMI (HR: 0.62, 95%CI: 0.3 to 1.3, *p* = 0.21) [12].

### 3.5. All-Cause Mortality

**Figure 4:**
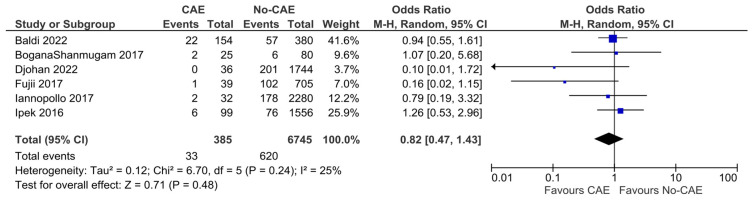
Forest plot of odds ratio (OR) for all-cause mortality in patients with AMI and evidence of CAE versus no-CAE. Results from Mantel–Haenszel (M-H) random-effects meta-analysis, comparing patients with CAE versus patients without CAE. CIs = Confidence intervals [11,12,20,22,23,25].

**Figure 5:**
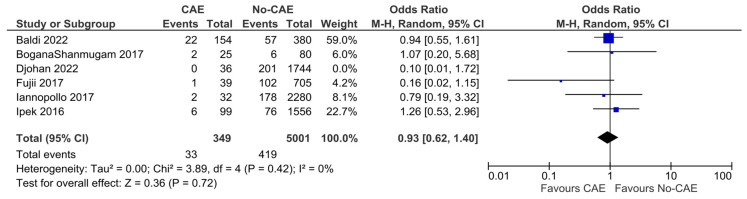
Post hoc sensitivity analysis on all-cause mortality odds, including only studies that enrolled patients with evidence of ectasia solely in the infarct-related vessel. Results from Mantel–Haenszel (M-H) random-effects meta-analysis, comparing patients with CAE versus patients without CAE. CIs = Confidence intervals [11,12,20,22,23,25].

The mortality rates ranged between zero and 14.3% in the study groups, and between 4.8% and 14.5% in the control groups. In the pooled analysis of six studies, CAE was not associated with significantly higher all-cause mortality, compared to patients with non-ectatic coronary vessels (OR: 0.82, 95% CI: 0.47 to 1.43, *p* = 0.48). There was moderate evidence of heterogeneity (χ^2^ = 6.70, I^2^= 25%) (Figure 4). In a sensitivity analysis, incorporating only the five studies that included patients with evidence of ectasia solely in the infarct-related artery, the results were similar (OR: 0.93, 95% CI: 0.62 to 1.40, χ^2^ =3.89, I^2^= 0%) (Figure 5).

### 3.6. Cardiac Death

**Figure 6:**
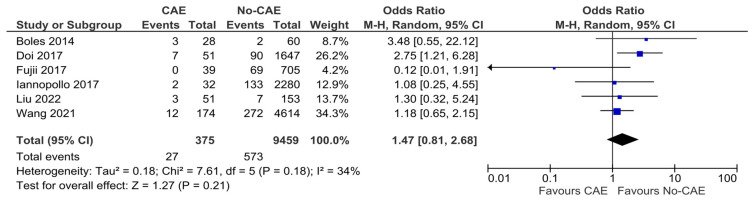
Forest plot of odds ratios (ORs) for cardiac death in patients with AMI and evidence of CAE versus No-CAE. Results from Mantel–Haenszel (M-H) random-effects meta-analysis, comparing patients with CAE versus patients without CAE. CIs = Confidence intervals [10,21,23,24,25,26].

Six of the included studies reported the rate of mortality due to cardiac-related causes. The pooled analysis did not yield results significantly different between patients with CAE and without CAE presenting with AMI (OR: 1.47, 95% CI: 0.81 to 2.68, *p* = 0.21, χ^2^ = 7.61, I^2^ = 34%) (Figure 6).

### 3.7. Repeat Myocardial Infarction

**Figure 7:**
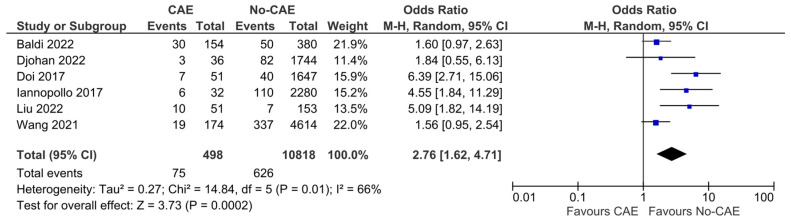
Forest plot of odds ratios (ORs) for repeat myocardial infarction in patients with AMI and evidence of CAE versus no CAE. Results from Mantel–Haenszel (M-H) random-effects meta-analysis, comparing patients with CAE versus patients without CAE. CIs = Confidence intervals [12,21,22,24,25,26].

**Figure 8:**
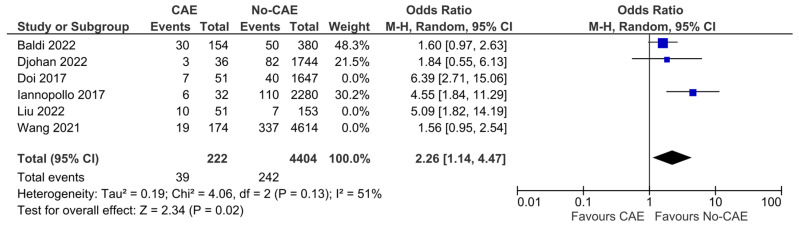
Post hoc sensitivity analysis on repeat myocardial infarction odds, including studies that enrolled only patients with STEMI. Results from Mantel–Haenszel (M-H) random-effects meta-analysis, comparing patients with CAE versus patients without CAE. CIs = Confidence intervals [12,21,22,24,25,26].

Regarding the risk of recurrent AMI, the reported outcomes of six studies and a total number of 11,316 patients (CAE group: 498, no-CAE group: 10,818) were pooled. The patients with CAE were found to have 2.76-fold higher odds of suffering from a recurrent AMI, compared to patients without CAE (OR:2.76, 95% CI:1.62 to 4.71, *p* < 0.001) (Figure 7). The presence of CAE was associated with an increased absolute risk of repeat AMI of 8.6% (95% CI 3.2% to 16.4%), corresponding to an NNH of 12 (95% CI 6 to 31). The results, however, presented significant heterogeneity (χ^2^ = 14.84, I^2^ = 66%). In the sensitivity analysis, including studies that enrolled patients with STEMI patients solely, the results were consistent, with remaining significant heterogeneity (OR: 2.26, 95% CI: 1.14 to 4.47, χ^2^ = 4.06, I^2^ = 51%) (Figure 8).

### 3.8. Repeat Revascularization

**Figure 9:**
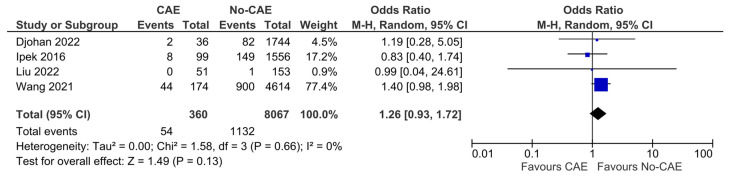
Forest plot of odds ratios (ORs) for repeat revascularization in patients with AMI and evidence of CAE versus no CAE. Results from Mantel–Haenszel (M-H) random-effects meta-analysis, comparing patients with CAE versus patients without CAE. CIs = Confidence intervals [11,12,24,26].

The pooled analysis of four cohorts that reported the overall effect of CAE on the necessity for repeat coronary revascularization showed that patients with CAE did not have higher odds for repeat revascularization, compared to controls (OR:1.26, 95% CI: 0.93 to 1.72, *p* = 0.13, χ^2^ = 1.58, I^2^ = 0%) (Figure 9).

### 3.9. Stroke

**Figure 10:**
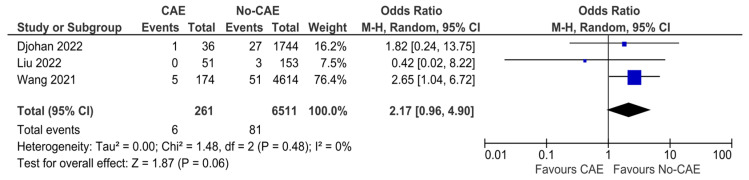
Forest plot of odds ratios (ORs) and funnel plot for stroke events in patients with AMI and evidence of CAE versus no CAE. Results from Mantel–Haenszel (M-H) random-effects meta-analysis, comparing patients with CAE versus patients without CAE. CIs = Confidence intervals [12,24,26].

Three studies reported stroke events as an outcome between patients with CAE and patients without CAE. The estimated effect of CAE in future stroke events was not significant (OR: 2.17, 95% CI: 0.96 to 4.90, *p* = 0.06, χ^2^ = 1.48, I^2^ = 0%) (Figure 10).

### 3.10. Reporting Bias

The visual inspection of funnel plots suggested asymmetry; however, given the small number of studies, formal assessment of publication bias is unreliable, and these findings should be interpreted cautiously (Supplementary Figures S1–S6).

## 4. Discussion

This systematic review and meta-analysis demonstrate that patients with AMI who exhibit CAE on angiography have significantly higher odds of experiencing MACEs compared to those without CAE. Importantly, the primary endpoint of the meta-analysis was primarily driven by a significantly higher incidence of recurrent myocardial infarction in the CAE group compared to the non-CAE group. This could be partially explained by the variations in event definitions or limited statistical power for less frequent outcomes, such as all-cause and cardiac mortality, in the studies’ follow-up duration.

Patients with CAE undergoing PCI, particularly in the context of AMI, may be at a higher risk compared to those without CAE. Ectasia in the infarct-related artery introduces several technical challenges during PCI. Stent placement can be difficult due to the abnormal size and geometry of the ectatic vessel, increasing the risk of stent malposition. This can lead to areas of turbulent blood flow, which may promote thrombus formation and, consequently, an increased risk of recurrent myocardial infarction [27]. However, the proposed mechanisms remain hypothetical and require confirmation in prospective studies. In cases of diffuse CAE, incomplete revascularization is also a concern [28]. Untreated significant ectatic segments remain susceptible to future adverse events [29].

Another potential mechanism for recurrent MI in this population is the progression of atherosclerosis and thrombosis in non-stented ectatic segments. While PCI addresses specific atherosclerotic lesions, the remaining ectatic areas are still vulnerable to atherosclerotic progression and thrombosis due to altered blood flow dynamics [29]. This creates a heightened risk for new thrombus formation or atherosclerotic plaque rupture. Furthermore, even after successful PCI, persistent inflammation in CAE can contribute to ongoing plaque instability or thrombosis, increasing the likelihood of recurrent MI [30].

The observed difference between the primary analysis and the STEMI-only sensitivity analysis regarding MACEs as a composite outcome suggests that population composition may influence the estimated effect size. This is not unexpected, given the pathophysiological differences between STEMI and NSTEMI, including variations in clinical characteristics and treatment strategies [31,32]. However, the attenuation of the effect in the STEMI-only analysis may reflect the reduced sample size, rather than a true absence of association.

Patients with CAE are at higher rates among males and smokers, predisposing for higher risk of adverse events. This could potentially lead to an overestimation of the association between coronary ectasia and MACEs. Moreover, greater operator volume and experience have been consistently associated with better procedural outcomes in complex procedures, acting as an unmeasured confounder [33]. This is of particular significance in CAE, where procedural heterogeneity and technical difficulties may further enhance operator-dependent variability.

In another meta-analysis conducted by Eid et al., no association was found between CAE and MACEs [15]. This discrepancy between their findings and those of the present study may be attributed to the more sensitive methodology used in this analysis, which included a larger number of studies. However, the evidence regarding AMI did not extend to a reproducible association with the need for recurrent revascularization procedures. Despite this, significantly higher rates of revascularization were observed in the CAE group within the largest cohort study included in the analysis [23]. Interestingly, this particular study reported much higher revascularization rates compared to others, though the authors were unable to explain the cause of this divergence.

### Limitations

The results of our systematic review and meta-analysis should be interpreted in the context of several limitations. Firstly, the definition of MACEs in the included studies varied significantly, including different combinations of outcomes. Among the missing outcomes across the studies, the final reported articles might be products of significant selective reporting bias. Additionally, all studies were observational and mostly retrospective in design, making the results of this review prone to selection bias. Most studies differed regarding the population, as only two of them enrolled patients with both STEMI and NSTEMI. Also, in most studies, patients with CAE were not matched with controls for potentially significant baseline characteristics and comorbidities. Finally, in some of our pooled synthesis results, the heterogeneity of the studies was significant, reducing the weight of our results.

## 5. Conclusions

The present study has determined that the available evidence supports that CAE is associated with an increased risk for future MACEs in patients with AMI. Patients with AMI and angiographic evidence of CAE face a higher risk of recurrent myocardial infarction compared to those without coronary ectasia. The risks associated with all-cause mortality, cardiac death, and repeat revascularization were found to be similar between patients with and without CAE. Future large-scale and well-organized studies are expected to shed light on the risks that CAE predisposes for future MACEs in patients with AMI.

## Figures and Tables

**Table 1:** Summary of included studies on major adverse cardiovascular events in patients with AMI and CAE.

Authors; Year	Type of Study	Setting	Participants			Mean Follow-Up (Months)	Type of AMI	Inclusion of CAE Other than EIRA	Reported Outcomes
			CAE	No-CAE	Total				
**Boles et al.; 2014 [10]**	Retrospective cohort study	UK	28	60	88	24	STEMI	N/A	- MACEs (ACS, repeat AMI, cardiac death) - Cardiac death
**Bogana Shanmugam et al.; 2017 [20]**	Retrospective cohort study	Australia	25	80	105	36	STEMI	No	- MACEs (All-cause mortality, repeat AMI, unstable angina, need for CABG) - All-cause mortality
**Ipek et al.; 2016 [11]**	Retrospective cohort study	Turkey	99	1556	1665	12	STEMI	No	- All-cause mortality - Repeat revascularization
**Iannopollo et al.; 2017 [25]**	Prospective cohort study	Italy	32	2280	2312	12	STEMI	No	- MACEs (All-cause mortality, repeat AMI) - All-cause mortality - Cardiac death - Repeat AMI
**Doi et al.; 2017 [21]**	Retrospective cohort study	Japan	51	1647	1698	49	Both STEMI and NSTEMI	Yes	- MACEs (Cardiac death, nonfatal AMI) - Cardiac death - Repeat AMI
**Djohan et al.; 2022 [12]**	Retrospective cohort study	Singapore	36	1744	1780	36	STEMI	Yes	- MACEs (All-cause mortality, repeat AMI, repeat revascularization, stroke, heart failure) - All-cause mortality - Repeat AMI - Repeat revascularization - Stroke - Heart failure
**Baldi et al.; 2022 [22]**	Retrospective cohort study	Italy	154	380	534	40	STEMI	Yes	- All-cause mortality - Repeat AMI
**Fujii et al.; 2017 [23]**	Retrospective cohort study	Japan	39	705	744	27	STEMI	No	- All-cause mortality - Cardiac death
**Wang et al.; 2021 [24]**	Retrospective cohort study	The Netherlands	174	4614	4788	48	Both STEMI and NSTEMI	Yes	- MACEs (Cardiac death, repeat AMI, repeat revascularization, stroke) - Cardiac death - Repeat AMI - Repeat revascularization - Stroke
**Liu et al.; 2022 [26]**	Retrospective Cohort Study	China	51	153	204	60	Both STEMI and NSTEMI	Yes	- MACEs (Cardiac death, repeat AMI, repeat revascularization, stroke, major hemorrhagic events, malignant arrhythmias, cardiogenic shock, readmission) - Cardiac death - Repeat AMI - Repeat revascularization - Stroke

CAE: coronary artery ectasia, AMI: acute myocardial infarction, EIRA: ectatic infarct-related artery, STEMI: ST-elevation myocardial infarction, NSTEMI: non-STEMI, MACEs: major adverse cardiovascular events, ACS: acute coronary syndrome, CABG: coronary artery bypass graft, N/A: not available.

**Table 2:** The baseline characteristics and comorbidities of the participants in the included studies.

Authors; Year	Age (Years)			Gender (Male)			STEMI at Presentation			Diabetes			Hypertension			Dyslipidemia			Smoking		
	CAE	No-CAE	*p*-Value	CAE	No-CAE	*p*-Value	CAE	No-CAE	*p*-Value	CAE	No-CAE	*p*-Value	CAE	No-CAE	*p*-Value	CAE	No-CAE	*p*-Value	CAE	No-CAE	*p*-Value
**Boles et al.; 2014 [10]**	-	-	-	-	-	-	All	All	-	3/30 (10.0%)	19/60 (30.7%)	**0.02**	13/30 (43.4%)	22/60 (36.6%)	0.82	17/30 (56.7%)	22/60 (36.7%)	0.65	24/30 (80.0%)	34/60 (56.7%)	0.43
**Bogana Shanmugam et al.; 2017 [20]**	52.8 ± 14.6	56.5 ± 9.8	0.35	88.0%	97.5%	0.16	All	All	-	0/25 (0.0%)	21/80 (26.3%)	**0.01**	10/25 (40.0%)	33/80 (41.3%)	0.91	8/25 (32.0%)	32/80 (40.0%)	**0.01**	16/25 (64.0%)	58/80 (72.5%)	0.57
**Ipek et al.; 2016 [11]**	58.0 ± 20.0	58.0 ± 17.0	0.91	86.8%	81.0%	0.09	All	All	-	26/99 (26.3%)	521/1556 (33.5%)	0.08	52/99 (52.5%)	602/1556 (38.7%)	**0.005**	36/99 (36.4%)	543/1556 (34.9%)	0.42	49/99 (49.5%)	508/1556 (32.6%)	**0.001**
**Iannopollo et al.; 2017 [25]**	65.9 ± 11.6	62.9 ± 12.5	0.17	81.3%	77.8%	0.637	All	All	-	7/32 (21.9%)	374/2280 (16.7%)	0.44	-	-	-	-	-	-	-	-	-
**Doi et al.; 2017 [21]**	63.0 ± 13.0	68.0 ± 12.0	0.005	84.3%	71.2%	**0.04**	43/51 (84.3%)	1318/1647 (80.0%)	0.45	15/51 (29.4%)	652/1647 (39.6%)	0.14	38/51 (74.5%)	1086/1647 (65.9%)	0.20	24/51 (47.1%)	896/1647 (54.4%)	0.30	44/51 (86%	1163/1647 (71%)	**0.02**
**Djohan et al.; 2021 [12]**	57.1 ± 11.7	58.1 ± 12.2	0.444	91.7%	87.2%	0.61	All	All	-	4/36 (11.1%)	1548/1744 (31.4%)	**0.01**	16/36 (44.4%)	902/1744 (51.7%)	0.41	18/36 (50.0%)	1060/1744 (60.8%)	0.23	17/36 (47.2%)	841/1744 (48.2%)	**0.04**
**Baldi et al.; 2020 [22]**	64.6 ± 12.0	62.3 ± 13.7	0.069	90.9%	72.6%	**<0.001**	All	All	-	18/154 (11.7%)	98/380 (25.8%)	**0.001**	98/154 (63.6%)	231/380 (60.8%)	0.06	64/154 (41.6%)	160/380 (42.1%)	**0.01**	111/154 (72.1%)	237/380 (62.4%)	0.21
**Fujii et al.; 2017 [23]**	56.3 ± 11.4	66.8 ± 12.4	<0.001	87.2%	78.0%	0.18	All	All	-	9/39 (23.1%)	260/705 (36.9)	0.08	30/39 (76.9%)	537/705 (76.2%)	0.91	28/39 (71.8%)	493/705 (69.9%)	0.80	17/39 (43.6%)	255/705 (36.2%)	0.16
**Wang et al.; 2021 [24]**	62.0 ± 12	63.0 ± 12.0	0.766	81.6%	73.6%	**0.02**	158/174 (90.8%)	4215/4614 (91.4%)	0.801	12/174 (6.9%)	608/4614 (13.2%)	0.05	58/174 (33.3%)	1780/4614 (38.6%)	0.32	112/174 (64.4%)	2777/4614 (60.2%)	0.24	105/174 (60.3%)	2395/4614 (51.9%)	0.09
**Liu et al.; 2022 [26]**	63.0 (55.0 to 74.0)	62.0 (55.0 to 75.0)	0.948	80.4%	77.8%	0.95	NA	NA	NA	16/51 (31.4%)	49/153 (32%)	0.93	30/51 (58.8%)	89/153 (58.2%)	0.94	NA	NA	NA	31/51 (60.8%)	85/153 (55.6%)	0.51

**Table 3:** Angiographic characteristics of included vessels.

Authors; Year	Infarct in the Ectatic Vessel	Ectasia Related Vessels	Infarct Related Vessels		
			CAE	No-CAE	*p*-Value
**Boles et al.; 2014 [10]**	-	-	-	-	-
**Bogana Shanmugam et al.; 2017 [20]**	All	LAD: 8/25 (32%)	LAD: 8/25 (32%)	LAD: 33/80 (41.3%)	0.55
		RCA 12/25 (48%)	RCA 12/25 (48%)	RCA: 43/80 (53.8)	0.05
		LCx: 12/25 (48%)	LCx: 12/25 (48%)	LCx: 4/80 (5%)	0.79
**Ipek et al.; 2016 [11]**	All	LAD: 29.3%	LAD: 29.3%	-	-
		RCA: 45.5%,	RCA: 45.5%		
		LCx: 25.3%	LCx: 25.3%		
**Iannopollo et al.; 2017 [25]**	All	RCA: 16/32 (50%)	RCA: 16/32 (50%)	RCA: 739/2280 (32.4%)	**0.035**
**Doi et al.; 2017 [21]**	29/51 (56.9%)	LAD: 22/51 (43.1%)	LAD: 19/51 (37.3%)	LAD:791/1647 (48.0%)	-
		RCA: 39/51 (76.4%)	RCA: 21/51 (41.2%)	RCA: 585/1647 (35.6%)	-
		LCx: 28/51 (54.9%)	LCx: 10/51 (19.6%)	LCx: 219/1647 (13.3%)	-
**Djohan et al.; 2022 [12]**	35/36 (97.2%)	LAD: 16/36 (44.4%)	LAD: 9/36 (25%)	LAD: 834/1744 (47.8%)	**0.003**
		RCA: 25/36 (69.4%)	RCA: 23/36 (88.5%)	RCA: 670/1744 (38.4%)	**0.04**
		LCx: 6/36 (16.7%)	LCx: 4/36 (11.1%)	LCx: 206/1744 (18.1%)	0.330
**Baldi et al.; 2022 [22]**	89/154 (57.8%)	LAD: 62/154 (40.3%)	-	-	-
		RCA: 122/154 (79.2%)			
		LCx: 54/154 (35.1%)			
**Fujii et al.; 2017 [23]**	All	LAD: 7/39 (17.9%)	LAD: 7/39 (17.9%)	LAD: 328/705 (46.5%)	-
		RCA: 27/39 (69.2%)	RCA: 27/39 (69.2%)	RCA: 273/705 (38.7%)	**0.0029**
		LCx: 4/39 (10.3%)	LCx: 4/39 (10.3%)	LCx: 57/705 (8.0%)	-
**Wang et al.; 2021 [24]**	139/174 (79,9%)	LAD: 115/174 (66.1%)	LAD: 57/174 (32.8%)	LAD: 1886/4614 (40.9%)	**0.03**
		RCA: 138/174 (79.3%)	RCA: 72/174 (41.8%)	RCA:1639/4614 (35.5%)	0.11
		LCx: 90/174 (51.7%)	LCx: 29/174 (16.7%)	LCx: 703/4614 (15.2%)	0.61
**Liu et al.; 2022 [26]**	NA	LM: 3/51 (5.7%)	LM: 0/51 (0.0%)	LM: 0/153 (0.0%)	
		LAD: 14/51 (26.4%)	LAD: 4/51 (7.8%)	LAD: 23/153 (15.0%)	
		RCA: 34/51 (64.1%)	RCA: 16/51 (31.4%)	RCA: 66/153 (43.1%)	
		LCx: 17/51 (32.0%)	LCx: 6/51 (11.8%)	LCx: 21/153 (13.7%)	

## Data Availability

The data extracted from the included studies, along with the analysis code, are available from the corresponding author upon request.

## Supplementary Materials

The following supporting information can be downloaded at: https://www.mdpi.com/article/10.3390/jcm15093482/s1, Table S1: The Newcastle–Ottawa Quality Assessment Scale (NOS) of the included studies on an outcome level; Table S2: A summary of findings for primary and secondary outcomes presented in the included studies; Figure S1: Funnel plot of odds ratios (ORs) for additional MACEs in patients with AMI and evidence of CAE versus no CAE; Figure S2: Funnel plot of odds ratios (ORs) for all-cause mortality in patients with AMI and evidence of CAE versus no CAE.; Figure S3: Funnel plot of odds ratios (ORs) for cardiac death in patients with AMI and evidence of CAE versus no CAE; Figure S4: Funnel plot of odds ratios (ORs) for repeat myocardial infarction in patients with AMI and evidence of CAE versus no CAE.; Figure S5: Funnel plot of odds ratios (ORs) for repeat revascularization in patients with AMI and evidence of CAE versus no CAE.; Figure S6: Funnel plot of odds ratios (ORs) for stroke events in patients with AMI and evidence of CAE versus no CAE.; Supplementary S1: MEDLINE via PubMed Search String and Strategy; Supplementary S2: CENTRAL Search Strategy; Supplementary S3: Scopus Search String; Supplementary S4: PRISMA 2020 Checklist.

## Abbreviations

The following abbreviations are used in this manuscript:

aHR	Adjusted hazard ratio
AMI	Acute myocardial infarction
CA	Coronary angiography
CABG	Coronary artery bypass graft
CAD	Coronary artery disease
CAE	Coronary artery ectasia
CCTA	Coronary computed tomography angiography
CIs	Confidence Intervals
GRADE	Grading of Recommendations Assessment, Development and Evaluation
HR	Hazard ratio
LAD	Left anterior descending branch
LCx	Left circumflex coronary artery
MACEs	Major adverse cardiovascular events
MINOCAs	Myocardial infarction with nonobstructive coronary arteries
NOS	Newcastle–Ottawa Quality Assessment Scale
NNH	Number needed to harm
NSTEMI	Non-ST-elevation myocardial infarction
OR	Odds ratio
PCI	Percutaneous coronary intervention
PRESS EBC	Evidence Based Checklist for the Peer Review of Electronic Search Strategies
PRISMA	Preferred Reporting Items for Systematic Reviews and Meta-Analysis
RCA	Right coronary artery
STEMI	ST-elevation myocardial infarction
