# Supplementary material for: Major Adverse Cardiovascular Events in Patients with Acute Myocardial Infarction and Angiographic Evidence of Coronary Artery Ectasia: A Systematic Review and Meta-Analysis
Source: J Clin Med. 2026 May 1;15(9):3482. doi: 10.3390/jcm15093482 (PMC13163636; doi:10.3390/jcm15093482)
Supplement: Supplementary file 1 [file jcm-15-03482-s001.zip › jcm-4232870-supplementary.pdf]

SUPPLEMENTARY FILES

TABLES

1. **Supplementary Table S1. N**ewcastle-Ottawa Quality Assessment Scale (NOS) of the included studies on an outcome level.
2. **Supplementary Table S2.** Summary of findings for primary and secondary outcomes presented in the included studies.

FIGURES

1. **Supplementary Figure S1** Funnel plot of odds ratios (OR) for additional MACE in patients with AMI and evidence of CAE versus No-CAE.
2. **Supplementary Figure S2** Funnel plot of odds ratios (OR) for all-cause mortality in patients with AMI and evidence of CAE versus No-CAE.
3. **Supplementary Figure S3** Funnel plot of odds ratios (OR) for cardiac death in patients with AMI and evidence of CAE versus No-CAE
4. **Supplementary Figure S4** Funnel plot of odds ratios (OR) for repeat myocardial infarction in patients with AMI and evidence of CAE versus No-CAE.
5. **Supplementary Figure S5** Funnel plot of odds ratios (OR) for repeat revascularization in patients with AMI and evidence of CAE versus No-CAE.
6. **Supplementary Figure S6** Funnel plot of odds ratios (OR) for stroke events in patients with AMI and evidence of CAE versus No-CAE.

Supplementary

1. **Supplementary S1.** MEDLINE via PubMed Search String and Strategy
2. **Supplementary S2.** CENTRAL Search Strategy
3. **Supplementary S3.** Scopus Search String
4. **Supplementary S4.** PRISMA Checklist

Supplementary Table S1. Newcastle-Ottawa Quality Assessment Scale (NOS) of the included studies on an outcome level.

Studies	Selection<br>(*) Representativeness<br>(*) Selection of non-exposed cohort<br>(*) Ascertainment of exposure<br>(*) Outcome not present at the start	Comparability<br>(**) Based on the design or analysis	Outcome<br>(*) Assessment of the outcome<br>(*) Duration of follow-up<br>(*) Adequacy of follow-up	Total score
		**Major Adverse Cardiovascular Events**		
**Boles et al. 2014**	****	**	**	8
**Bogana Shanmugam et al. 2017**	****	**	***	9
**Doi et al. 2017**	****	-	***	7
**Djohan et al. 2022**	****	-	***	7
**Wang et al. 2021**	****	-	***	7
**Liu et al. 2022**	****	**	**	8
		**All-cause Mortality**		
**Bogana Shanmugam et al. 2017**	****	**	***	9
**Ipek et al. 2016**	****	-	***	7
**Iannopollo et al. 2017**	****	-	***	7
**Djohan et al. 2022**	****	-	***	7
**Baldi et al. 2022**	****	-	***	7
**Fujii et al. 2017**	****	-	**	6
		**Cardiac Death**		
**Boles et al. 2014**	****	**	**	8
**Iannopollo et al. 2017**	****	-	***	7
**Doi et al. 2017**	****	-	***	7
**Fujii et al. 2017**	****	-	**	6
**Wang et al. 2021**	****	-	***	7
**Liu et al. 2022**	****	**	**	8
		**Acute Myocardial Infarction**		
**Iannopollo et al. 2017**	****	-	***	7

**Doi et al. 2017**	****	-	***	7
**Djohan et al. 2022**	****	-	***	7
**Baldi et al. 2022**	****	-	***	7
**Wang et al. 2021**	****	-	***	7
**Liu et al. 2022**	****	**	**	8
**Repeat Revascularization**				
**Ipek et al. 2016**	****	-	***	7
**Djohan et al. 2022**	****	-	***	7
**Wang et al. 2021**	****	-	***	7
**Liu et al. 2022**	****	**	**	8
**Stroke**				
**Djohan et al. 2022**	****	-	***	7
**Wang et al. 2021**	****	-	***	7
**Liu et al. 2022**	****	**	**	8
**Heart Failure**				
**Djohan et al. 2022**	****	-	***	7

Supplementary Table S2. Summary of findings for primary and secondary outcomes presented in the included studies.

**Authors; Year**	**MACE**			**All-Cause Mortality**			**Cardiac Death**			**Repeat AMI**			**Repeat Revascularization**			**Stroke**			**Heart Failure**		
	**CAE**	**No-CAE**	**p-value**	**CAE**	**No-CAE**	**p-value**	**CAE**	**No-CAE**	**p-value**	**CAE**	**No-CAE**	**p-value**	**CAE**	**No-CAE**	**p-value**	**CAE**	**No-CAE**	**p-value**	**CAE**	**No-CAE**	**p-value**
**Boles et al.; 2014**	8/28 (28.5%)	13/60 (21.7%)	0.42	-	-	-	3/28 (10.7%)	2/60 (3.3%)	0.15	-	-	-	-	-	-	-	-	-	-	-	-
**Bogana Shanmugam et al.; 2017**	11/25 (44%)	13/80 (16.3%)	**0.01**	2/25 (8.0%)	6/80 (7.5%)	0.93	-	-	-	-	-	-	-	-	-	-	-	-	-	-	-
**Ipek et al.; 2016**	-	-	-	6/99 (6.0%)	76/1556 (4.8%)	0.37	-	-	-	-	-	-	8/99 (8.0%)	149/1556 (9.6%)	0.39	-	-	-	-	-	-
**Iannopollo et al.; 2017**	8/32 (26.2%)	263/2280 (12.7%)	**0.026**	2/32 (6.3%)	178/2280 (7.8%)	0.72	2/32 (6.3%)	133/2280 (8.5%)	0.722	6/32 (18.8%)	110/2280 (4.8%)	**<0.001**	-	-	-	-	-	-	-	-	-
**Doi et al.; 2017**	14/51 (27.5%)	130/1647 (7.9%)	**<0.001**	-	-	-	7/51 (13.7%)	90/1647 (5.5%)	**0.004**	7/51 (13.7%)	40/1647 (2.4%)	**<0.001**	-	-	-	-	-	-	-	-	-

**Djohan et al.; 2021**	5/36 (13.9%)	386/1744 (22.1%)	0.24	0/201 (0.0%)	36/1744 (11.5%)	**0.028**	-	-	-	3/36 (8.3%)	82/1744 (4.7%)	0.25	2/36 (5.6%)	82/1744 (4.7%)	0.686	1/36 (2.7%)	27/1744 (1.5%)	0.428	2/36 (5.6%)	103/1744 (5.9%)	1.000
**Baldi et al.; 2020**	-	-	-	22/154 (14.3%)	57/380 (15%)	0.93	-	-	-	30/154 (19.5%)	50/380 (13.2%)	0.09	-	-	-	-	-	-	-	-	-
**Fujii et al.; 2017**	-	-	-	1/39 (2.6%)	102/705 (14.5%)	**0.03**	0/39 (0.0%)	69/705 (9.8%)	**0.04**	-	-	-	-	-	-	-	-	-	-	-	-
**Wang et al.;2021**	64/174 (36.7%)	1181/4614 (25.6%)	**<0.001**	-	-	-	12/174 (6.9%)	272/4614 (5.9%)	0.54	19/174 (10.9%)	337/4614 (7.3%)	**0.006**	44/174 (25.3%)	900/4614 (19.5%)	**0.03**	5/174 (2.8%)	51/4614 (1.1%)	**0.03**	-	-	-
**Liu et al.;2022**	13/51 (25.5%)	18/153 (11.8%)	**0.018**	-	-	-	3/51 (5.9%)	7/153 (4.6%)	0.71	10/51 (19.6%)	7/153 (4.6%)	**<0.001**	0/51 (0.0%)	1/153 (0.7%)	0.99	0/51 (0.0%)	3/153 (2.0%)	0.57			

CAE: coronary artery ectasia, AMI: acute myocardial infarction, MACE: major adverse cardiovascular events

Da ta are presented as n: / N (%)

Level of significance: a=0.05

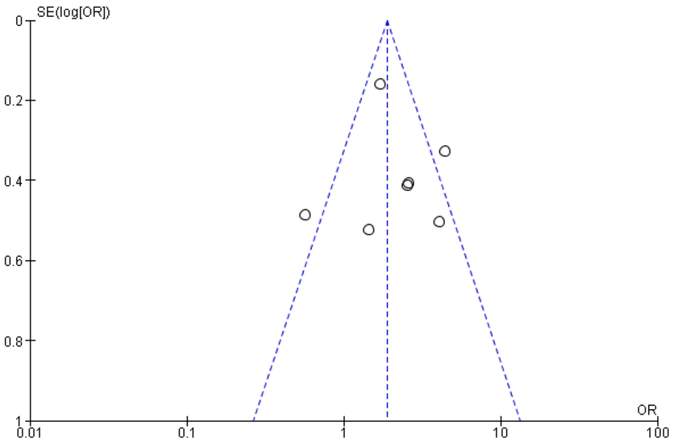

Supplementary Figure S1. Funnel plot of odds ratios (OR) for additional MACE in patients with AMI and evidence of CAE versus No-CAE

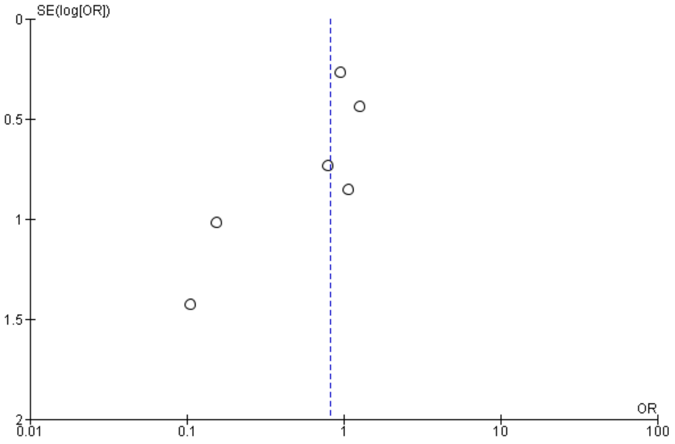

Supplementary Figure S2. Funnel plot of odds ratios (OR) for all-cause mortality in patients with AMI and evidence of CAE versus No-CAE

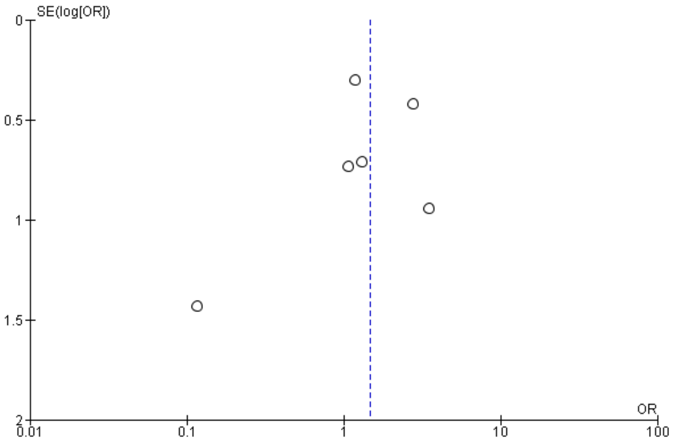

Supplementary Figure S3. Funnel plot of odds ratios (OR) for cardiac death in patients with AMI and evidence of CAE versus No-CAE

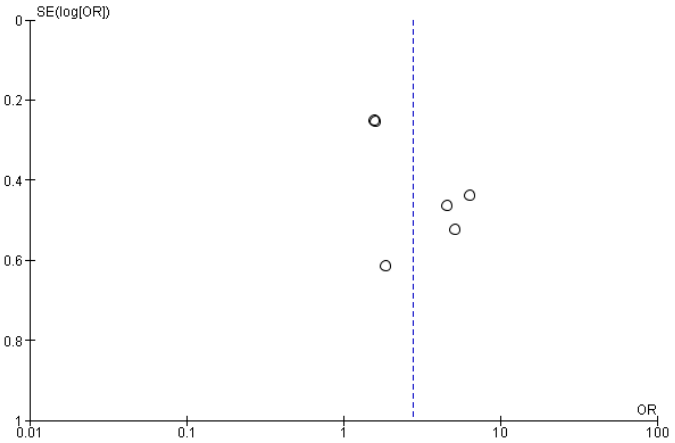

Supplementary Figure S4. Funnel plot of odds ratios (OR) for repeat myocardial infarction in patients with AMI and evidence of CAE versus No-CAE

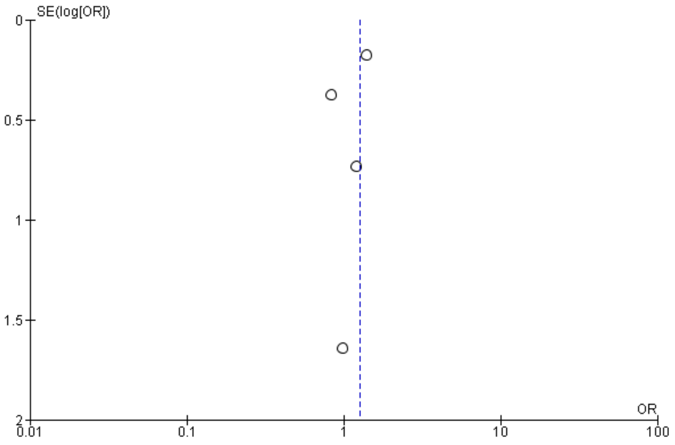

Supplementary Figure S5. Funnel plot of odds ratios (OR) for repeat revascularization in patients with AMI and evidence of CAE versus No-CAE

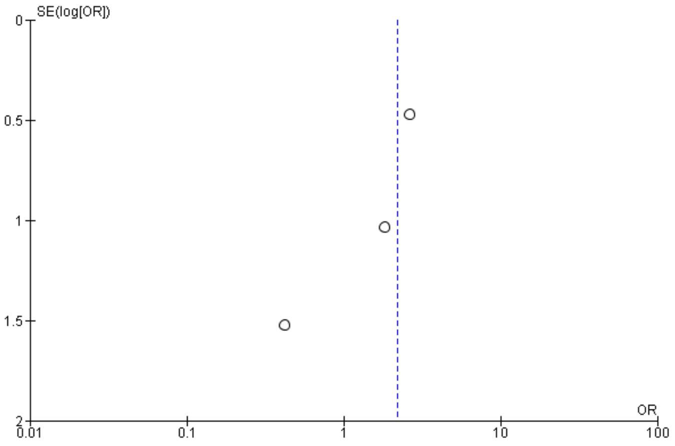

Supplementary Figure S6. Funnel plot of odds ratios (OR) for stroke events in patients with AMI and evidence of CAE versus No-CAE

Supplementary S1

MEDLINE via PubMed Search String

(((myocardial infarction) OR (myocardial infarction[MeSH Terms]) OR (non ST elevated myocardial infarction) OR (non ST elevated myocardial infarction[MeSH Terms]) OR (non-ST elevated myocardial infarction) OR (non ST elevation myocardial infarction) OR (non-ST elevated myocardial infarction) OR (non-STEMI) OR (NSTEMI) OR (ST elevated myocardial infarction[MeSH Terms]) OR (ST elevated myocardial infarction) OR (ST-elevated myocardial infarction) OR (ST elevation myocardial infarction) OR (ST-elevation myocardial infarction)) AND ((coronary angiography[MeSH Terms]) OR (coronary angiography) OR (percutaneous coronary intervention[MeSH Terms]) OR (percutaneous coronary intervention) OR (PCI) OR (angioplasty[MeSH Terms]) OR (angioplasty))) AND ((coronary ectasia) OR (coronary aneurysm) OR (aneurysm, coronary[MeSH Terms]) OR (coronary aneurysms[MeSH Terms]))

MEDLINE via PubMed Search Strategy

Myocardial Infarction

#1: myocardial infarction [All Fields]

#2: myocardial infarction [MeSH Terms]

#3: non ST elevated myocardial infarction [All Fields]

#4: non ST elevated myocardial infarction [MeSH Terms]

#5: non-ST elevated myocardial infarction [All Fields]

#6: non ST elevation myocardial infarction [All Fields]

#7: non-ST elevated myocardial infarction [All Fields]

#8: non-STEMI [All Fields]

#9: NSTEMI [All Fields]

#10: ST elevated myocardial infarction [MeSH Terms]

#11: ST elevated myocardial infarction [All Fields]

#12: ST-elevated myocardial infarction [All Fields]

#13: ST elevation myocardial infarction [All Fields]

#14: ST-elevation myocardial infarction [All Fields]

#15: #1 OR #2 OR #3 OR #4 OR #5 OR #6 OR #7 OR #8 OR #9 OR #10 OR #10 OR #11 OR #12 OR #13 OR #14

Coronary Angiography

#16: coronary angiography [MeSH Terms]

#17: coronary angiography [All Fields]

#18: percutaneous coronary intervention [MeSH Terms]

#19: percutaneous coronary intervention [All Fields]

#20: PCI [All Fields]

#21: angioplasty [MeSH Terms]

#22: angioplasty [All Fields]

#23: #16 OR #17 OR #18 OR #19 OR #20 OR #21 OR #22

Myocardial Infarction undergoing Coronary Angiography

#24: #15 AND #23

Coronary ectasia

#25: coronary ectasia [All Fields]

#26: coronary aneurysm [All Fields]

#27: aneurysm, coronary [MeSH Terms]

#28: coronary aneurysms [MeSH Terms]

#29: #25 OR #26 OR #27 OR #28

Final Search: #24 AND #29

Supplementary S2

CENTRAL Search Strategy

ID	Search	Hits
#1	(myocardial infarction)	35028
#2	MeSH descriptor: [Myocardial Infarction] explode all trees	11858
#3	(non ST elevated myocardial infarction)	417
#4	(non-ST elevated myocardial infarction)	284
#5	(non ST elevation myocardial infarction)	2823
#6	(non-ST elevation myocardial infarction)	1869
#7	(non-STEMI)	130
#8	(NSTEMI)	721
#9	(ST elevated myocardial infarction)	664

#10 (ST-elevated myocardial infarction) 195

#11 (ST elevation myocardial infarction) 7042

#12 (ST-elevation myocardial infarction) 3563

#13 #1 OR #2 OR #3 OR #4 OR #5 OR #6 OR #7 OR #8 OR #9 OR #10 OR #11 OR #12 35171

#14 MeSH descriptor: [Coronary Angiography] explode all trees 4506

#15 (coronary angiography) 10580

#16 MeSH descriptor: [Percutaneous Coronary Intervention] explode all trees 6151

#17 (percutaneous coronary intervention) 12175

#18 (PCI) 9873

#19 MeSH descriptor: [Angioplasty, Balloon, Coronary] explode all trees 3550

#20 (angioplasty) 9787

#21 #14 OR #15 OR #16 OR #17 OR #18 OR #19 OR #20 27302

#22 (coronary ectasia) 20

#23 (coronary aneurysm) 592

#24 MeSH descriptor: [Coronary Aneurysm] explode all trees 36

#25 #22 OR #23 OR #24 608

#26 #13 AND #21 AND #25 126

Supplementary S3

Scopus Search String

(TITLE-ABS-KEY (myocardial AND infarction) AND TITLE-ABS-KEY ((coronary AND angiography)) AND TITLE-ABS-KEY ((coronary AND ectasia) OR (coronary AND aneurysm)))

Supplementary S4

PRISMA 2020 Checklist

Section and Topic	Item #	Checklist item	Location where item is reported
**TITLE**			
Title	1	Identify the report as a systematic review.	p.1, lines 2-4
**ABSTRACT**			
Abstract	2	See the PRISMA 2020 for Abstracts checklist.	p.1, lines 14-35
**INTRODUCTION**			
Rationale	3	Describe the rationale for the review in the context of existing knowledge.	p.2, lines 44-59
Objectives	4	Provide an explicit statement of the objective(s) or question(s) the review addresses.	p.2, lines 60-62
**METHODS**			
Eligibility criteria	5	Specify the inclusion and exclusion criteria for the review and how studies were grouped for the syntheses.	p.2-3, lines 71-95
Information sources	6	Specify all databases, registers, websites, organisations, reference lists and other sources searched or consulted to identify studies. Specify the date when each source was last searched or consulted.	p.2-3, lines 97-108
Search strategy	7	Present the full search strategies for all databases, registers and websites, including any filters and limits used.	Supplementary file, Appendices A,B,C
Selection process	8	Specify the methods used to decide whether a study met the inclusion criteria of the review, including how many reviewers screened each record and each report retrieved, whether they worked independently, and if applicable, details of automation tools used in the process.	p.3, lines 113-124
Data collection process	9	Specify the methods used to collect data from reports, including how many reviewers collected data from each report, whether they worked independently, any processes for obtaining or confirming data from study investigators, and if applicable, details of automation tools used in the process.	p. 3-4, lines 126-132
Data items	10a	List and define all outcomes for which data were sought. Specify whether all results that were compatible with each outcome domain in each study were sought (e.g. for all measures, time points, analyses), and if not, the methods	p. 3, lines 86-95

Section and Topic	Item #	Checklist item	Location where item is reported
		used to decide which results to collect.	
	10b	List and define all other variables for which data were sought (e.g. participant and intervention characteristics, funding sources). Describe any assumptions made about any missing or unclear information.	p.3, lines 86-95
Study risk of bias assessment	11	Specify the methods used to assess risk of bias in the included studies, including details of the tool(s) used, how many reviewers assessed each study and whether they worked independently, and if applicable, details of automation tools used in the process.	p. 4, lines 134-142
Effect measures	12	Specify for each outcome the effect measure(s) (e.g. risk ratio, mean difference) used in the synthesis or presentation of results.	p. 4, lines 146-148
Synthesis methods	13a	Describe the processes used to decide which studies were eligible for each synthesis (e.g. tabulating the study intervention characteristics and comparing against the planned groups for each synthesis (item #5)).	p. 4, lines 148-150
	13b	Describe any methods required to prepare the data for presentation or synthesis, such as handling of missing summary statistics, or data conversions.	p. 4, lines 147-148
	13c	Describe any methods used to tabulate or visually display results of individual studies and syntheses.	p. 4, line 152
	13d	Describe any methods used to synthesize results and provide a rationale for the choice(s). If meta-analysis was performed, describe the model(s), method(s) to identify the presence and extent of statistical heterogeneity, and software package(s) used.	p. 4, lines 150-155 and 161-163
	13e	Describe any methods used to explore possible causes of heterogeneity among study results (e.g. subgroup analysis, meta-regression).	p. 4 lines 156-160
	13f	Describe any sensitivity analyses conducted to assess robustness of the synthesized results.	p. 4, lines 159-160
Reporting bias assessment	14	Describe any methods used to assess risk of bias due to missing results in a synthesis (arising from reporting biases).	p. 4, lines 165-168
Certainty assessment	15	Describe any methods used to assess certainty (or confidence) in the body of evidence for an outcome.	NA
**RESULTS**			
Study selection	16a	Describe the results of the search and selection process, from the number of records identified in the search to the number of studies included in the review, ideally using a flow diagram.	p. 5, lines 171-185
	16b	Cite studies that might appear to meet the inclusion criteria, but which were excluded, and explain why they were excluded.	p. 5, Figure 1
Study characteristics	17	Cite each included study and present its characteristics.	p.5-8, lines 187 - 235
Risk of bias in studies	18	Present assessments of risk of bias for each included study.	p. 8-9 lines 236-252
Results of individual studies	19	For all outcomes, present, for each study: (a) summary statistics for each group (where appropriate) and (b) an effect estimate and its precision (e.g. confidence/credible interval), ideally using structured tables or plots.	Supplmentary Table S2
Results of syntheses	20a	For each synthesis, briefly summarise the characteristics and risk of bias among contributing studies.	p. 9 line 270-271, p.10 lines 307-308, p. 11 line 337

Section and Topic	Item #	Checklist item	Location where item is reported
	20b	Present results of all statistical syntheses conducted. If meta-analysis was done, present for each the summary estimate and its precision (e.g. confidence/credible interval) and measures of statistical heterogeneity. If comparing groups, describe the direction of the effect.	p. 9 lines 267-278, p. 10 lines 303-363
	20c	Present results of all investigations of possible causes of heterogeneity among study results.	p. 9, lines 273-274, p. 11, lines 340-342
	20d	Present results of all sensitivity analyses conducted to assess the robustness of the synthesized results.	p.10, 308-311
Reporting biases	21	Present assessments of risk of bias due to missing results (arising from reporting biases) for each synthesis assessed.	p. 12, lines 366-368
Certainty of evidence	22	Present assessments of certainty (or confidence) in the body of evidence for each outcome assessed.	NA
**DISCUSSION**			
Discussion	23a	Provide a general interpretation of the results in the context of other evidence.	
	23b	Discuss any limitations of the evidence included in the review.	p.12-13, lines 373-403
	23c	Discuss any limitations of the review processes used.	p. 13, lines 405-415
	23d	Discuss implications of the results for practice, policy, and future research.	p.13, lines 422-423
**OTHER INFORMATION**			
Registration and protocol	24a	Provide registration information for the review, including register name and registration number, or state that the review was not registered.	p. 2, lines 65-69
	24b	Indicate where the review protocol can be accessed, or state that a protocol was not prepared.	p. 2, line 69
	24c	Describe and explain any amendments to information provided at registration or in the protocol.	NA
Support	25	Describe sources of financial or non-financial support for the review, and the role of the funders or sponsors in the review.	p. 14, line 441
Competing interests	26	Declare any competing interests of review authors.	p.14,line 447
Availability of data, code and other materials	27	Report which of the following are publicly available and where they can be found: template data collection forms; data extracted from included studies; data used for all analyses; analytic code; any other materials used in the review.	p. 14, lines 445-447

From: Page MJ, McKenzie JE, Bossuyt PM, Boutron I, Hoffmann TC, Mulrow CD, et al. The PRISMA 2020 statement: an updated guideline for reporting systematic reviews. BMJ 2021;372:n71. doi: 10.1136/bmj.n71. This work is licensed under CC BY 4.0. To view a copy of this license, visit https://creativecommons.org/licenses/by/4.0/
